# Experiences in a Coagulation Referral Clinic in Bristol

**Published:** 1985-01

**Authors:** Helena M. Daly, G. Wakerley

**Affiliations:** Department of Haematology, Bristol Royal Infirmary; Department of Haematology, Bristol Royal Infirmary


### Experiences in a Coagulation Referral Clinic in Bristol

**Helena M. Daly, M.R.C.P.I., M.R.C.Path.**
Senior Registrar in Haematology
**G. Wakerley, F.I.M.L.S.**
Chief M.L.S.O. in Coagulation,
Department of Haematology, Bristol Royal Infirmary

### THE NEED FOR A COAGULATION REFERRAL CLINIC

A seven year old boy underwent routine tonsillectomy. The operation was complicated by catastrophic haemorrhage. The screening tests—Prothrombin Time (PT) and Activated Partial Thromboplastin Time (APTT) were normal. He was treated empirically with fresh frozen plasma but bleeding continued. Bilateral carotid ligation was ultimately required to arrest haemorrhage.

He was subsequently referred to this hospital for investigation. We elicted from his mother a history of easy bruising, menorrhagia and post-partum haemorrhage. The boy also had a history of bruising. However, he had undergone adenoidectomy at four years without excessive blood loss. Both he and his mother were found to have von Willebrand's Disease.

Patients with severe inherited coagulation defects rarely present a diagnostic problem. They bleed spontaneously and present in early childhood. It is mildly affected patients who may remain undiagnosed. They seldom bleed spontaneously. However, extensive bleeding may occur following surgical or traumatic challenge. The investigation of such patients in this hospital has been haphazard in the past. Patients attended for blood tests without prior consultation with Haematologists. As a result, in many cases inadequate or inappropriate samples were taken. This was the reason for setting up the Coagulation Referral Clinic and this paper describes the results of the first year.

### ORGANISATION OF THE CLINIC

The clinic is held once per week. The average number of patients seen is four. Patients are advised not to take aspirin-containing compounds for ten days prior to investigation to avoid interference with platelet aggregation studies. Each patient is examined and has a detailed bleeding history taken, with particular reference to the incidence of epistaxis, bruising, menorrhagia, and blood loss following surgery, dental extraction or other trauma.

The basic coagulation screen consists of a bleeding time (Ivy), which must be performed by an experienced person to provide meaningful results, platelet count, prothrombin time (PT), activated partial thromboplastin time (APTT), and thrombin clotting time (TCT). In addition the following assays are performed—Factor VIII Coagulant activity (Factor VIII C) to detect Haemophilia A; Factor IX to detect Haemophilia B; and Factor VIII Related Antigen (Factor VIII RAG) and Factor VIII Ristocetin Co-Factor (Factor VIII RiCoF) which are essential for the diagnosis of von Willebrand's Disease. These investigations require considerable technical expertise and are not available in every hospital. Other specific factor assays are performed when indicated by the history and screening tests. Platelet function studies (Aggregation and ADP, Collagen, Ristocetin and Arachadonic Acid) and total platelet nucleotides (ADP and ATP) are performed when the bleeding time is prolonged (greater than 10 minutes) or when indicated by the history.

All patients receive a letter stating whether they are normal or abnormal. A more detailed letter is sent to the referring doctor and a copy to the patient's notes. Patients diagnosed as having a coagulation defect are registered at the Haemophilia Centre and issued with a Special Medical Card describing their defect. They are counselled on the significance of the disorder, need for replacement therapy prior to surgery and the need to screen other family members.

### PATIENTS

Ninety-nine new patients were referred in 1983 (58 female and 41 male) with an age range from 3–78 years (Mean 30.2 years). Eighty-nine per cent of the patients were diagnosed within two visits. The source of referral is shown in Table 1. Twelve patients were referred for confirmation of a diagnosis made many years ago. Eleven patients were referred for investigation of a possible carrier state for Haemophilia. Twenty-one patients (25%) were investigated directly by us as part of a family study. Other referral symptoms are shown in Table 2. Many patients had more than one symptom. Only 28.6% of the patients found to be normal required more than one visit compared to 40% of those subsequently diagnosed as abnormal.

The type of coagulation defect detected are listed in Table 3.

**Table 1**

**Source of Referral**

| | |
|---|---|
| Family study | 21 |
| Dental surgeon | 19 |
| General practitioner | 16 |
| ENT surgeon | 10 |
| Paediatrician | 9 |
| Haematologist | 9 |
| Gynaecologist | 5 |
| Surgeons | 6 |
| Physicians | 4 |

**Table 2**

**Referral Symptoms**

| | |
|---|---|
| Bled post dental extractions | 26 |
| Family history of bleeding disorder (Diagnosis known) | 22 |
| Bruising | 20 |
| Menorrhagia | 14 |
| Carrier detection | 11 |
| Confirmation of a previous diagnosis | 12 |
| Family history of bleeding (Diagnosis unknown) | 12 |
| Post tonsillectomy | 9 |
| Post other surgery | 7 |
| Epistaxis | 6 |
| Thrombocytosis | 3 |
| Abnormal bleeding from cuts | 3 |
| Haemarthrosis | 2 |
| Post partum haemorrhage | 1 |
| Rectal bleeding | 1 |
| Haemorrhagic tonsilitis | 1 |
| Threatened abortion | 1 |
| Traumatic haematoma | 1 |
| Labial haematoma | 1 |

### DISCUSSION

The case described at the beginning of this article highlights several problems related to patients with coagulation defects. The previous bleeding history may be mild and fail to draw the attention of the doctor to a possible bleeding disorder. Mildly affected patients may bleed following one operation and not after another, this particularly true of von Willebrand's Disease. In such cases bleeding is most likely to occur when surgery on mucosal surfaces is performed. However, when bleeding does occur in an unsuspected case, it may be life-threatening.

**Table 3**

**Abnormal Results**

| | |
|---|---|
| von Willebrand's disease | 11 |
| Platelet function defects | 5 |
| Mild haemophilia A (Factor VIII 5–35%) | 5 |
| Moderate haemophilia A (Factor VIII 1–5%) | 3 |
| Mild factor VII deficiency (Same family) | 3 |
| Factor VIII carrier | 3 |
| Factor IX carrier | 3 |
| Mild haemophilia B (Factor IX 5–35%) | 2 |
| Hypercoaguable state | 2 |

A detailed history of previous haemostatic challenge is essential, but not always taken. The classical textbook description of joint bleeding in coagulation defects and mucosal bleeding in platelet defects, only applies to severe coagulation defects. It does not hold true for patients who are mildly affected, and considerable attention must be paid to the history in an attempt to differentiate between normal and excessive blood loss. When the history is suggestive of abnormal bleeding the patient should be referred pre-operatively for investigation. This would avoid cancelling operations, the associated psychological trauma and needless expense. Many patients are referred to us too late to have the necessary investigations performed in time for surgery and one patient had had her pre-med before the sample arrived in the laboratory.

The most common disorder detected was von Willebrand's Disease. This reflects the changing pattern of diagnosis due to the availability of more sophisticated tests (Factor VIII RAG and Factor VIII RiCoF) to differentiate this disorder from Haemophilia A. Two patients in this series diagnosed as having mild Haemophilia A in the early 1970's were re-diagnosed on the basis of these investigations as having von Willebrand's Disease. Inherited disorders of platelet function are rare and difficult to classify. There was only one clear diagnosis in this series— a patient with storage pool deficiency associated with oculo-cutaneous albinism (Hermansky-Pudlak syndrome). The other four patients had non-specific but persistent defects of secondary platelet aggregation.

Eleven patients were referred for investigation of possible carrier status for Haemophilia A or B. Two were excluded on the basis of family history alone; six were confirmed as being carriers and in two cases the carrier state could neither be established nor excluded with the techniques available. This is an important function of the clinic as this problem creates a considerable amount of anxiety amongst female relatives of Haemophiliacs. Only one patient in this series with a significant history of bleeding had no abnormality found on detailed laboratory investigations. However, this does highlight the limitations of the investigations available at this time.

We feel this clinic is useful. 31% of patients seen had a significant defect detected. This is most important now as appropriate replacement therapy is available to prevent haemorrhage in all of these conditions. Many of these patients would not be detected by a routine coagulation screen (PT, APTT, BT and platelet count) as the sensitivity of these tests is such that they may fail to detect mild cases. Consequently if the history is suggestive of a significant coagulation defect specific factor assays must be performed. These patients must be referred for investigation on an elective basis. It is well documented that stress may increase the level of Factor VIII C in patients with moderately low levels. This is illustrated by a three year old boy in this series who was diagnosed as having mild Haemophilia A (Factor VIII C 25%). He was re-referred because of bleeding disproportional to his Factor VIII level and non-accidental injury had been suspected. When re-tested he was found to have a Factor VIII level of 4% which was in keeping with the clinical situation. This particular problem is encountered with nervous or young patients.

As yet we have made no attempt to extend this service to the investigation of patients with hyper-coaguable states. The two patients diagnosed in this series were originally referred because of rectal bleeding (1) and bruising (1). Both were subsequently found to have advanced malignant disease. In general, patients presenting with thrombotic complications represent an unrewarding group of patients to investigate, as in the majority of cases no correctable abnormality can be found.

